# Protein diffusion from microwells with contrasting hydrogel domains

**DOI:** 10.1063/1.5078650

**Published:** 2019-04-19

**Authors:** Elaine J. Su, Shaheen Jeeawoody, Amy E. Herr

**Affiliations:** 1 Department of Bioengineering, University of California, Berkeley, Berkeley, California 94720, USA.; 2 UC Berkeley–UCSF Graduate Program in Bioengineering, Berkeley, California 94720, USA; 3 Chan Zuckerberg Biohub, San Francisco, California 94158, USA

## Abstract

Understanding and controlling molecular transport in hydrogel materials is important for biomedical tools, including engineered tissues and drug delivery, as well as life sciences tools for single-cell analysis. Here, we scrutinize the ability of microwells—micromolded in hydrogel slabs—to compartmentalize lysate from single cells. We consider both (i) microwells that are “open” to a large fluid (i.e., liquid) reservoir and (ii) microwells that are “closed,” having been capped with either a slab of high-density polyacrylamide gel or an impermeable glass slide. We use numerical modeling to gain insight into the sensitivity of time-dependent protein concentration distributions on hydrogel partition and protein diffusion coefficients and open and closed microwell configurations. We are primarily concerned with diffusion-driven protein loss from the microwell cavity. Even for closed microwells, confocal fluorescence microscopy reports that a fluid (i.e., liquid) film forms between the hydrogel slabs (median thickness of 1.7 *μ*m). Proteins diffuse from the microwells and into the fluid (i.e., liquid) layer, yet concentration distributions are sensitive to the lid layer partition coefficients and the protein diffusion coefficient. The application of a glass lid or a dense hydrogel retains protein in the microwell, increasing the protein solute concentration in the microwell by ∼7-fold for the first 15 s. Using triggered release of Protein G from microparticles, we validate our simulations by characterizing protein diffusion in a microwell capped with a high-density polyacrylamide gel lid (p > 0.05, Kolmogorov-Smirnov test). Here, we establish and validate a numerical model useful for understanding protein transport in and losses from a hydrogel microwell across a range of boundary conditions.

## INTRODUCTION

Molecular transport through hydrogels is important across a wide range of bioengineering systems, including tissue engineering, drug delivery, and single-cell assays. In hydrogels, diffusion of macromolecules from one phase to another (i.e., liquid to hydrogel) is hindered by thermodynamic partitioning. The equilibrium partition coefficient, *K_eq_*, is defined as the ratio of concentration of solute in the gel to that in liquid

$$K_{\mathrm{eq}}=\;\frac{C_{\mathrm{gel}}}{C_{\mathrm{liquid}}},$$ (1)

where *C* is the solute mass per volume, *C*_*gel*_ is the solute concentration in the hydrogel, and *C*_*liquid*_ is the solute concentration in the liquid phase.^1,2^ In the absence of attractive interactions (e.g., van der Waals forces or electrostatic interactions^3^ between the solute and the gel), the partition coefficient is described by Ogston's model, which depends on the polymer volume fraction, the chain radius, and the size and shape of the solute molecule.^4^ Empirically, the partition coefficient of gels is quantitatively determined by measuring the relative concentration of a fluorescent species in the liquid phase and in the hydrogel phase for a given multimaterial system.^1,5,6^

In addition to equilibrium solute concentrations, time-dependent and diffusion-driven solute concentration gradients—both within a single material and between materials—are important. For example, understanding both drug delivery to the bloodstream and transport capabilities of cellular waste products out of capsules benefit from understanding these types of concentration distributions.^7–13^ Within homogeneous hydrogels such as polyacrylamide (PA), which have mobile polymer chains, solute diffusion in hydrogels behaves according to a scaled hydrodynamic model^10^ and can be empirically determined.^1,11^ In hydrogels with immobile polymer chains (e.g., alginate), the diffusion coefficient (*D*) of small molecular species can be described by a hydrogel obstruction model.^10^ When placed in a liquid bath, the transport of solute from the gel phase into the liquid phase can be characterized by using non-steady-state measurements. Using such approaches, *D* of small solute species was found to be 5%–50% lower in gel than in the surrounding water.^9^ The importance of the material type and properties in in-gel and out-of-gel diffusion has necessitated the development of methods to rapidly determine *D* of a solute in hydrogel systems.^14^

In general, the diffusion of a solute through a heterogeneous medium depends on the solubility and diffusivity of the solute in the different material domains and the geometry of the domains.^15^ Although various studies have reported on particle diffusion in locally heterogeneous hydrogels^16,17^ and in hybrid [e.g., Poly(ethylene oxide)-poly(acrylic acid) (PEO-PAA)] hydrogels,^18^ few studies have reported on solute diffusion through more than two different materials. *In vivo* systems are notably complex and are composed of multiple biological polymer networks (e.g., mucus, extracellular matrix).^16^ For example, the study of oxygen permeability through contact lenses to the cornea can be represented as two interfacing hydrogels. A fluid (i.e., liquid) film interface exists between these hydrogels; this interfacial layer varies in thickness according to the topography and morphology of the gel, interfacial tension, interface potential, adsorption, partitioning, and chemistry of the gel.^19^ Fluid films between hydrogels and human tissues, such as the cornea, can range from nanometers to tens of microns.^20–24^ Characterizing the fluid film thickness between sandwiched hydrogels is necessary for understanding molecular transport at the interface of the hydrogels.

Compartmentalization of cells in hydrogels has emerged as a useful approach for studying cellular processes. Hydrogel droplets encapsulating cells have facilitated biochemical analyses of individual cells.^25,26^ Similarly, encapsulation of cells in microwells allows researchers to scrutinize individual cells to study, in two examples, secreted proteins and nucleotides.^27,28^ In microwell-based studies, macromolecules diffuse from a cell and through free solution to react with antibody probes immobilized along the walls or the lid of the microwell,^29–32^ and the spatial location of the macromolecules (e.g., proteins) relative to the antibodies in the microwell can influence the detected signal strength.^33^

In our own research group, we have explored single-cell resolution protein electrophoresis using thin PA gels as the molecular sieving matrix. Our approach, called electrophoretic cytometry, isolates single cells in individual microwells. Cells are then chemically lysed in individual microwells, and the intracellular contents are subjected to electrophoresis in the hydrogel surrounding the microwell.^5,6,34^ To mitigate single-cell lysate diffusion out of the “open” microwell and electrophoresis gel, researchers have used a glass slide as a “lid” on the hydrogel structures. Capping with a glass lid improved lysate retention in the hydrogel.^35^ This particular study demonstrated that partitioning and material permeability can be modulated to maintain a high concentration of solute in a detection area.^30^ We have also demonstrated patterning chemistries onto the thin microwell-containing PA gel by applying a high density lid impregnated with the source chemistry, which concurrently mitigates diffusion of the single-cell lysate out of open microwells.^6,36^ Thus, understanding how time-dependent mass transport of proteins depends on operational parameters such as *K_eq_,* system geometries, and *D* provides a framework for design of bioanalytical tools, such as electrophoretic cytometry, with appreciable analytical sensitivity.

Here, we seek to understand the role of a lid layer in closing an open microwell used in electrophoretic cytometry, be that layer liquid, high-density PA gel, or glass. We have demonstrated patterning chemistries onto a thin layer of PA gel by applying a high-density lid impregnated with the source chemistry. We have also explored lid gels to mitigate diffusion of single-cell lysate out of open microwells.^6,36^ To understand the importance of each material and the role of thin fluid (i.e., liquid) layers that form between sandwiched hydrogels, we first characterize the thickness of fluid film layers that form between hydrogels of different densities. We then use our knowledge of fluid film thicknesses to create an experimentally validated numerical model that predicts the dependence of the microwell-encapsulated protein concentration on the fluid film thickness, partition coefficient of the hydrogels, and protein diffusivity in the lid gel for model proteins Green Fluorescent Protein (GFP) and Protein G. Understanding diffusion-driven transport of intracellular proteins in hydrogels and free solution ultimately aids selection of hydrogel properties in multimaterial systems, which should be useful for applications ranging from drug delivery to high-sensitivity diagnostics.

## RESULTS AND DISCUSSION

### Model of diffusion of GFP through heterogeneous materials

**FIG. 1.**
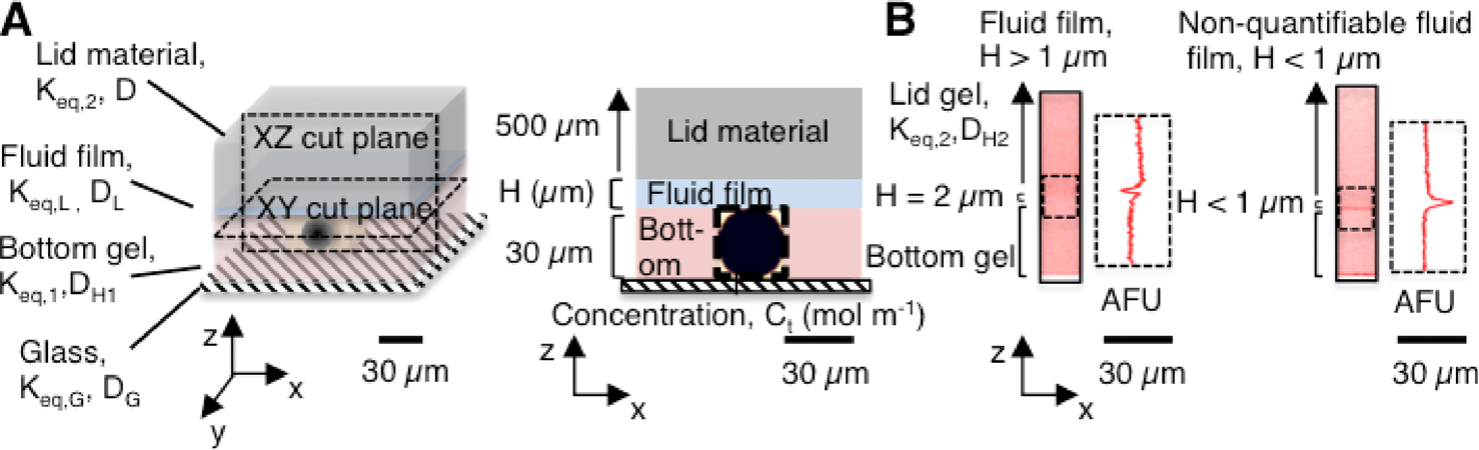
The fluid film is created by capping the bottom gel with a lid layer. (a) Schematic of the bottom gel (*K_eq_*_,1_), lid layer (Glass, *K_eq,G_* or lid gel, *K_eq,_*_2_), interfacial fluid film (thickness, *H*), and microwell housing a spherical protein source. The 30-um thick bottom gel houses a microwell (black dotted line) containing the spherical source of protein with an initial concentration, C_0_. Cut planes show side and top planes of the layered device, with the protein source material diffusing from the microwell and into surrounding materials over time. Over time, the diffusion coefficient (*D*) of protein in each surrounding material and *K_eq_* of each material determines diffusive losses of protein from the microwell into the respective material. (b) Confocal fluorescence micrographs (inverted, false color) of the fluid film created between the bottom gel and lid gel. The median measurable fluid film thickness was 1.7 *μ*m (n = 9), excluding fluid films lacking a quantifiable thickness (n = 8). PA, polyacrylamide; *K_eq_*, equilibrium partition coefficient; *D*, diffusion coefficient of GFP in each material.

We first sought to understand how protein lysate losses from the closed electrophoretic cytometry device are impacted by *K_eq_* and *D*. Lysate losses occur over time via diffusion and partitioning between the varying material and solution phases. Analytically, it is challenging to identify and quantify losses of lysate in varying geometries (domains) that comprise different material properties (and thus, varying partitioning and diffusion coefficients). Thus, we created and studied a 2D axisymmetric model (COMSOL) of a three-layer device [Fig. 1(a)] composed of: (1) a bottom gel, which is a 30-*μ*m thick, 6 %T PA gel conjugated onto a conventional microscope slide and which houses the 30-*μ*m diameter microwell; (2) a fluid film that arises at the interface of hydrated hydrogels and has a thickness *H*; and (3) a lid, which is 500 *μ*m thick and is composed of either a high-density (15 %T) PA hydrogel or glass. Material properties are provided in Table I, with *D* and *K_eq_* values obtained from the literature.^6^ The protein source was modeled as a 28-*μ*m diameter sphere. Upon release from the spherical source, the concentration of the protein solute in the microwell fluid volume (*C_t_*) decreases with time due to diffusion and chemical partitioning between the different material phases. With a large fluid reservoir, *C_t_* approaches *C*_*liquid*_ over time. We thus calculated GFP concentration distributions as a function of time. For demonstration purposes, the figures are labeled as Cartesian (x, y, z) coordinates rather than in cylindrical (r, θ, z) coordinates.

**TABLE I.** Partition and diffusion coefficient values for GFP in the range of layer materials characterized in this study.

Material name	Material composition	*K_eq_*	*D* (m^2^ s^–1^)
Glass lid	Glass	*K_eq,G_* = 0 (liquid, glass)	*D_G_* = 0
Bottom gel (hydrogel 1)	6 %T, 3.3 %C PA	*K_eq,_*_1_ = 0.51 (liquid, bottom gel)	*D_H_*_1_ = 3.13 × 10^–11^
Lid gel (hydrogel 2)	15 %T, 3.3 %C PA	*K_eq,_*_2_ = 0.24 (liquid, lid gel)	*D_H_*_2_ = 4.2 × 10^–12^
Liquid layer	Liquid	*K_eq,L_* = 1 (liquid, liquid)	*D_L_* = 1.691 × 10^–10^

To measure the fluid film thickness in a layered hydrogel device, we incorporated rhodamine methacrylate into the bottom gel and the lid gel (a 15 %T, 3.3 %C PA gel, where %T is the total amount of acrylamide and %C is the ratio of cross-linker mass to total monomer mass in the gel), incubated the sandwiched layers in a buffer solution (TBST) overnight, and then imaged the interface between the sandwiched hydrogels using confocal microscopy. We measured the thickness of the void between the bottom gel and the lid gel [Fig. 1(b)] using a method similar to that employed by Kuypers *et al.*^37^ Of the 17 total samples, 8 samples had no resolvable decrease in fluorescence in the void, and thus, the fluid film thickness was not quantifiable. We attribute the lack of a signal decrease to the possibility that these samples had fluid film thicknesses smaller than our z-axis resolution of 0.42 *μ*m, a resolution that is dictated by the pinhole diameter. Given the geometries of our system (tens to hundreds of micrometers), we considered the resolution acceptable. Of the quantifiable samples, the range of measured fluid layer thicknesses spanned from 1.3 to 1.9 *μ*m with a median fluid layer thickness of 1.7 *μ*m (n = 9). Our fluid layer thickness values are larger than the 300–600 nm fluid layer thickness values previously reported for permeable hydrogels;^38^ however, those reported values were for hydrogels with elastic moduli 3 orders of magnitude larger and much lower (44%) water content than the hydrogels considered here.^39^ Given that interfacial fluid films vary in thickness according to the topography, morphology, interfacial tension, interface potential, adsorption, partitioning, permeability, and chemistry of the gel,^19,40,41^ we anticipate a wide range of possible fluid film thicknesses, depending on the specific configuration of the hydrogel system under study. Our fluid layer thickness values fall in the range of fluid film thicknesses measured for layers that form between hydrogels and human tissues, such as the cornea.^20–24^

### Protein losses from closed and open microwells

**FIG. 2.**
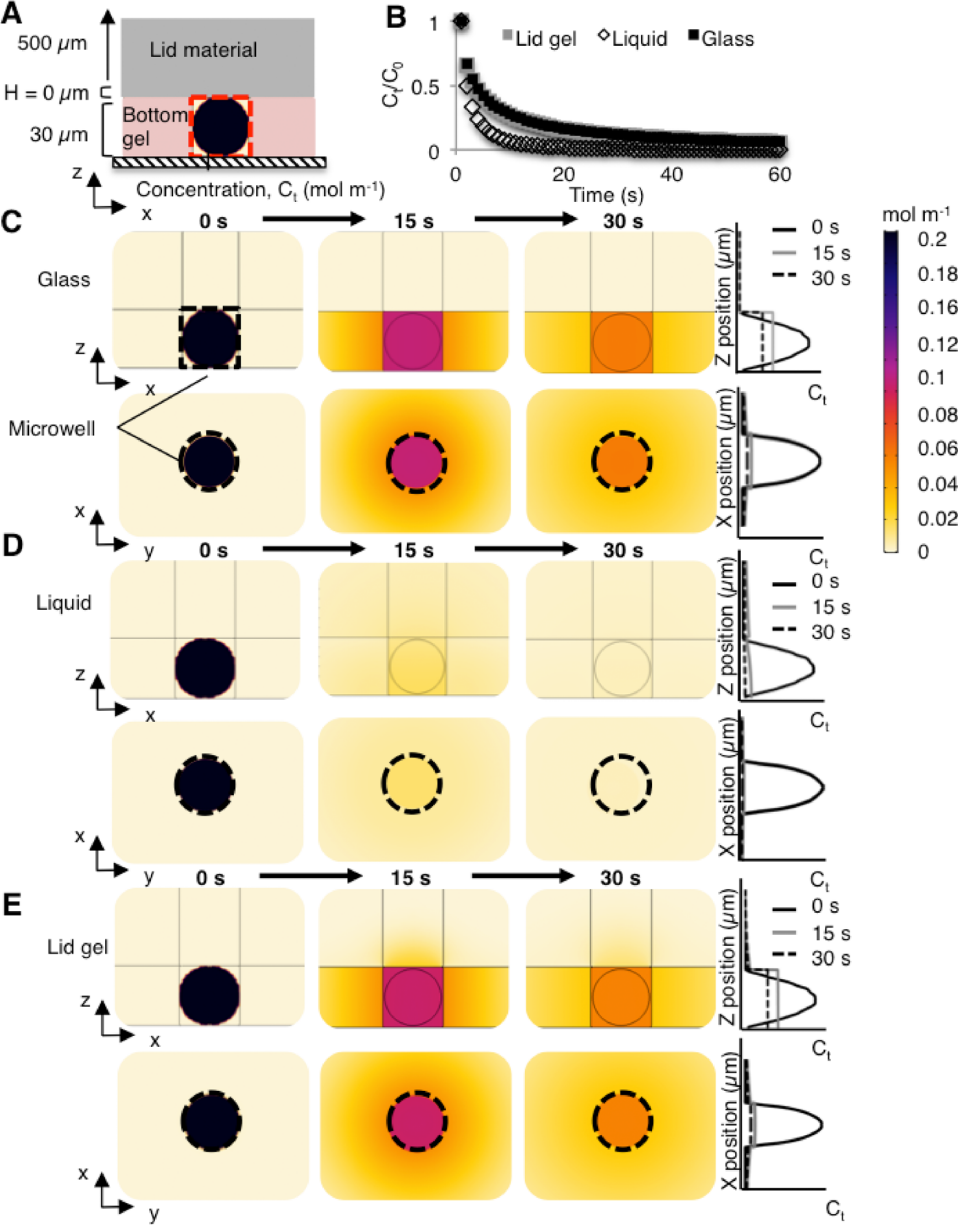
In the absence of a fluid layer, numerical simulations indicate that the lid material determines the concentration distribution of the protein source material diffusing out of the microwell. (a) Side-view schematic of the 2D-axisymmetric diffusion model for a range of lid materials (glass, liquid, or lid gel). (b) Comparison of the protein solute concentration in the microwell as a function of time, normalized to the initial protein solute concentration in the microwell, as a function of different lid materials. At time t = 15 s, the normalized protein solute concentration in the microwell, *C_t_*/*C*_0_, decreases to 0.20 when glass or a lid gel is used, compared to 0.03 in the liquid system. (c) A glass lid layer reduces diffusion in the z-axis. (d) A liquid lid layer (open microwell) leads to rapid diffusion-based dilution of the protein source material. (e) A lid gel mitigates diffusive losses as compared to the open configuration with the same thickness (*H *=* *500 *μ*m) shown in (d). The microwell is outlined in a dotted black line.

For a microwell capped with a lid (i.e., closed), we sought to characterize how *K_eq_* and *D* of GFP in the lid would affect the protein concentration in that microwell over time. Protein losses from the microwell occur as proteins diffuse and partition between the different media comprising the microwell (i.e., gel, liquid, and glass). We performed numerical simulations to determine the degree of protein retention in the microwell fluid volume, as a function of the lid material [Fig. 2(a)]. We opted to scrutinize three lid materials, based on our empirical systems; hence, the lid material was simulated as glass (assumed to be impermeable, *K_eq,G_* = 0, *D_G_* = 0 m^2^ s^−1^), liquid (i.e., free solution), or lid gel (dense gel). For each case, we determined the concentration of GFP in the microwell at every 1 s and normalized to the initial concentration [*C_t_*/*C*_0_, Fig. 2(b)].

With a closed microwell, *C_t_*/*C*_0_ increases compared to an open microwell (liquid bath as upper boundary condition) configuration. At time t = 15 s, a time point pertinent to cell lysis and protein solubilization in microwells,^5^ an open microwell configuration yields *C_t_*/*C*_0_= 0.03, whereas the presence of either a glass or lid gel yields *C_t_*/*C*_0_= 0.20 [Fig. 2(b)]. The application of a glass lid reduces diffusion of solute out of the microwell, since protein cannot partition into the lid, and the local solute concentration in the microwell fluid volume decreases with time owing to (i) dilution throughout the microwell volume and (ii) diffusion out of the microwell into the surrounding bottom gel [Fig. 2(c)]. We first estimated a characteristic diffusion time (*L*^2^/*D*) of ∼29 s for GFP to diffuse from the microwell into the bottom gel layer in the quiescent, closed microwell configuration (where *L* is the microwell diameter; *D* is the diffusion coefficient of GFP in the bottom gel). Using numerical simulation, we then assessed the protein concentration profiles at t = 0 s, 15 s, and 30 s, matching the time scales of cell lysis, protein solubilization, and the expected diffusion time scale.^5^ From the simulations, within 1 s, the GFP within the microwell becomes uniform. The concentration of GFP along the x-axis remains uniform at t = 15 s. The GFP concentration remains highest in the microwell fluid volume since partitioning inhibits protein from entering the surrounding medium (bottom gel) and since there is no transport into the impermeable glass lid.

In the configuration where the microwell is open to a reservoir of fluid, we modeled the free solution with *K_eq_*_,*L*_ = 1 and *D_L_* = 1.691 × 10^–10^ m^2^ s^−1^ [Fig. 2(d)]. The 500 *μ*m fluid layer thickness approximates a free solution bath, given the time scales of GFP diffusion.^6^ In our model, placing a glass lid 500  *μ*m away from the solute-containing microwell did not change the concentration distribution of protein in the microwell fluid volume, as compared to the 500 *μ*m free solution bath alone (data not shown). In contrast to the glass lid configuration, the protein concentration in the microwell fluid volume diminishes quickly for an open microwell (*C_t_*/*C*_0_ = 0.03 at t = 15 s), as expected. At t = 15 s, the protein concentration is highest at the bottom of the microwell since no flux occurs below the microwell into the glass support. *C_t_*/*C*_0_ diminishes to 0.01 by t = 30 s.

Next, we considered a hydrogel material as the lid layer. We investigated *C_t_*/*C*_0_ in the microwell fluid volume when a lid gel was applied [Fig. 2(e)]. In electrophoretic cytometry, high-density lid gels have been employed for diffusive delivery of reagents to the bottom gel and to mitigate out-of-plane diffusive losses from the microwell.^6,36,42,43^ Interestingly, *C_t_*/*C*_0_ was similar to that of the glass lid configuration [Figs. 2(b) and 2(e)]. We also investigated the solute concentration across the span of the bottom gel (i.e., the microwell and the entire bottom gel). *C_t_*/*C*_0_ of the bottom gel at t = 15 s was 0.96 when a lid gel was used, similar to *C_t_*/*C*_0_ = 1.0 calculated for the glass lid configuration.

Overall, the application of a lid layer (glass or high-density gel) that inhibits diffusion of protein solute into the lid layer is effective at maintaining high concentrations of solute in the microwell fluid volume. Future studies seek to understand how other physico-chemical properties may also be modulated to further improve solute retention in the microwell. For example, inclusion into the lid layer of interacting particles (e.g., via charge^16^ or hydrophilicity^44^) that bind to or obstruct proteins could further decrease the mass of solute that can enter the lid layer and preserve high solute concentrations in the microwell.

### Experimental validation of the model

**FIG. 3.**
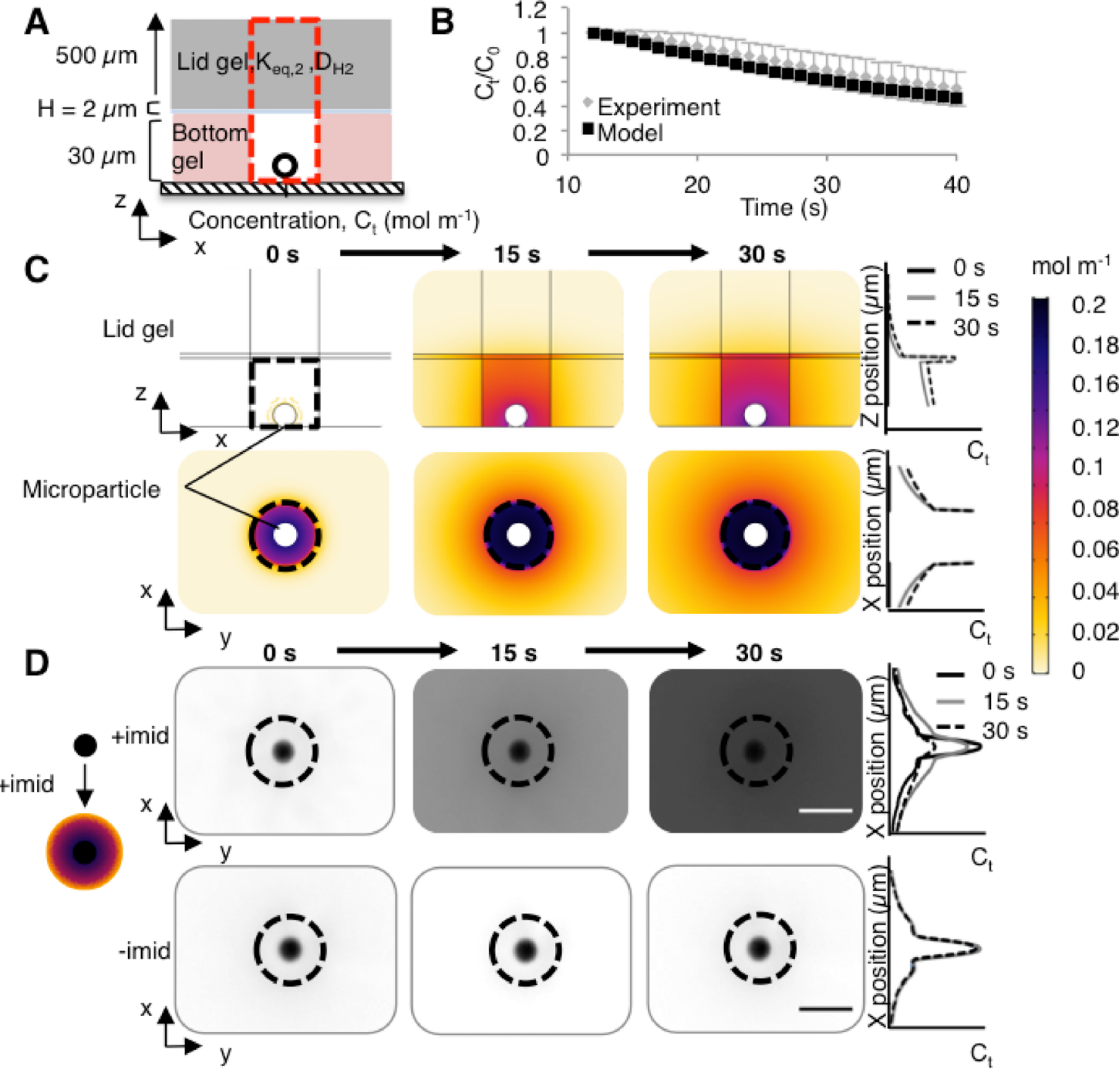
Experimental validation of protein release from a spherical source located in a hydrogel microwell. (a) Side-view schematic of the model and experiment, with 10-*μ*m diameter microparticles coated with fluorescently-labeled Protein G as the protein source (black circle). H = 2 *μ*m. (b) Comparison of simulations (model) and experiment reports similar (p > 0.05, Kolmogorov-Smirnov test) trends in normalized microwell fluorescence loss over time. For the simulations, an integral of the protein mass in the column extending from the microwell through the lid gel [red rectangle in (a)] was obtained to represent the mass of protein that is observed via wide-field microscopy (experiment). Error bars represent the standard deviation (n = 4). (c) Time series of simulation results show release of Protein G from a microparticle within a microwell (black dotted line). Protein G released per unit area was calculated using the number of molecules released per unit time, divided by the surface area of the microparticle. The number of Protein G molecules released per unit time was employed as a flux boundary condition in the simulations. (d) Time series of experimental results show inverted fluorescence micrographs of fluorescently-labeled Protein G release at t = 0, 15, and 30 s after the addition of imidazole (“+imid”), delivered by incubating the lid gel in a 1 M imidazole buffer solution. The negative control uses 0 M imidazole (“-imid”). The fluid layer thickness H is unknown in these experiments.

To experimentally validate our computational model of the electrophoretic cytometry device, we used a microparticle-based chemistry for rapid release of proteins from a spherical source in a microwell [Fig. 3(a)]. Microparticles (10-*μ*m diameter) conjugated with a Ni surface chemistry were coated with His-tagged proteins. Introduction of imidazole releases the protein from the particle surface, owing to competition between His and imidazole for Ni. We have developed the microparticles as a means to deliver protein size markers to each microwell in electrophoretic cytometry.^45^ Imidazole can be delivered using a lid gel, as previously described in single-cell electrophoretic assays.^6,36,42^ The Ni-His-imidazole release scheme gives short “switching” periods (seconds as compared to minutes or hours), appreciable release efficiency, and adequate spatial control for delivery to microwells, as compared to protein-PA conjugations,^46–48^ photo-labile polymers,^49–51^ caged particles,^52^ photo-activatable probes,^53^ drug-releasing nanogels,^54^ photo-assembly and photo-cleavable microcapsules,^55^ photo-controlled release micelles,^56^ protein-protein conjugations, and click chemistry. We use the median measured fluid film thickness of 2 *μ*m [Fig. 1(b)].

The kinetics of protein release from the microparticle source must be considered in constructing the simulations. To determine release kinetics, we first characterized the imidazole-triggered release of Alexa Fluor 647-labeled His-tagged Protein G (26 kDa) from microparticles. His-tagged Protein G is commercially available and has the same molecular mass as GFP. A magnet was used to actively settle Protein G-coated microparticles into microwells; excess microparticles were washed off the gel surface. The lid gel was incubated overnight in a 1 M imidazole solution. At t = 0 s, the lid layer was seated atop the bottom gel and the microparticle-laden microwells to initiate imidazole diffusion into the bottom gel and Protein G release from the microparticles. Epifluorescence microscopy was used to monitor the fluorescence intensity in the microwell fluid volume (Fig. S1), thus allowing normalization of fluorescence signal to the initial fluorescence intensity of the microparticle. For the fluorescence signal of the microparticles, we observed an exponential decay in fluorescence that matched the signal observed in our previously observed model of imidazole delivery using a convective delivery (pouring) system [y=1.2789exp(−0.035x)], R^2^ = 0.98, comparable to $y=1.18\exp\left(-0.04x\right)+0.08$ that was previously reported.^45^

Monitoring the fluorescence signal of the microparticle during Protein G release from the surface allowed us to establish a flux boundary condition for our simulations. First, we assumed the raw fluorescence intensity after background subtraction was equivalent to the number of fluorescent molecules or proteins. Since we ultimately normalize the final fluorescence signal to the initial signal, absolute quantification of fluorescent molecules is not necessary. Next, we defined a flux boundary condition for the microparticle protein source N(*t*) as

$$N\left(t\right)=\frac{d(\mathrm{Number}\;\mathrm{molecules}\;\left(t=0\right)-\mathrm{Number}\;\mathrm{molecules}\left(t\right))}{\mathrm{dt}}\times \frac{1}{4\pi r^{2}},$$ (2)

where *r* is the microparticle radius (5 × 10^–6^ m). We assume that *K_eq_* and *D* are similar for Protein G and GFP in the bottom gel, the liquid in the microwell fluid volume, and the lid gel, given the similarity in molecular mass. Advection from the lid gel placement was assumed to be negligible, as the Péclet number (Pe=uL/D, where *L* = 30 μm, *u* is the average measured velocity of the bead, 285 μm/s) was measured to be two orders of magnitude lower than with delivery of lysis buffer by pouring (n = 9 from 3 separate trials, Fig. S2). Moreover, we assumed that the microparticle was impermeable and that protein is conjugated only to the microparticle surface.

Comparison of experimental observations and simulations of the Protein G concentration in the microwell fluid volume showed reasonable agreement [p > 0.05, Kolmogorov-Smirnov test, Fig. 3(b)]. In our simulations, we measured the volume integral of Protein G mass in the column above the microwell to simulate observations via epifluorescence microscopy [Fig. 3(a), red dotted line]. Interestingly, in our experiments, we observed an initial increase in the fluorescence signal in the microwell fluid volume with the maximum measured fluorescence at t = 12 s [n = 4; Fig. S1(b)]. The increase in fluorescence is likely caused by self-quenching of the fluorescently-labeled protein while bound to the microparticle; at higher concentrations, fluorescent dyes can aggregate, causing quenching of up to 90% of the fluorescence signal until the dye molecules are spaced sufficiently far apart.^57^ We hypothesize that protein released from the microparticle surface is sufficiently diluted within the microwell such that self-quenching no longer occurs. For our simulations and our experiments, we thus normalized the concentration values to the maximum signal (i.e., at the 12 s time point).

In addition to the microwell volume, we also scrutinized the concentration distribution in the surrounding bottom gel, in the fluid film, and in the lid gel [Fig. 3(c)]. As expected, the highest concentration of Protein G (t = 15 s) was localized to the fluid film, which is a material that sees no partitioning-based exclusion of Protein G from the microwell fluid volume and which affords Protein G a high *D* as compared to within the hydrogels. Because *D* and *K_eq_* for Protein G are highest in the fluid film, the fluid volume rapidly accumulates protein. At the hydrogel walls of the microwell, we observe a Protein G concentration that drops off sharply, attributable to preferential partitioning of solute into the microwell fluid volume and *D* that is higher in the microwell fluid volume than in the surrounding hydrogel.

Both the simulation results and the experimental observations of solute signal released from microparticles indicated release of protein solute into the microwell fluid volume, with lower concentrations of protein solute in the surrounding hydrogel material [Fig. 3(d)]. While useful for illustrative purposes, two major caveats preclude direct quantitative comparison of the simulations and the experimental approximation. First, the observed concentration of the released solute is expected to be lower than in the XY plane of the simulations, as the XY plane in the simulations is taken through the center of the microwell (i.e., a cross-section), where the concentration of protein is maximal. In contrast, in the experiment, the entire volume of the microwell, including through the lid, is imaged. Second, the thickness of the fluid film was not measurable, as the temporal resolution of confocal microscopy exceeds the 0–30 s window of microparticle release. Nonetheless, the agreement observed between the experiment and simulations in the microwell volume [Fig. 3(b)] indicates that the simulations may be used to accurately predict protein concentrations within the microwell fluid volume for multiple materials, given *D* and *K_eq_* of a given protein into the lid material.

### Protein loss from microwells is dependent on *H*, *K_eq_*, and *D*

**FIG. 4.**
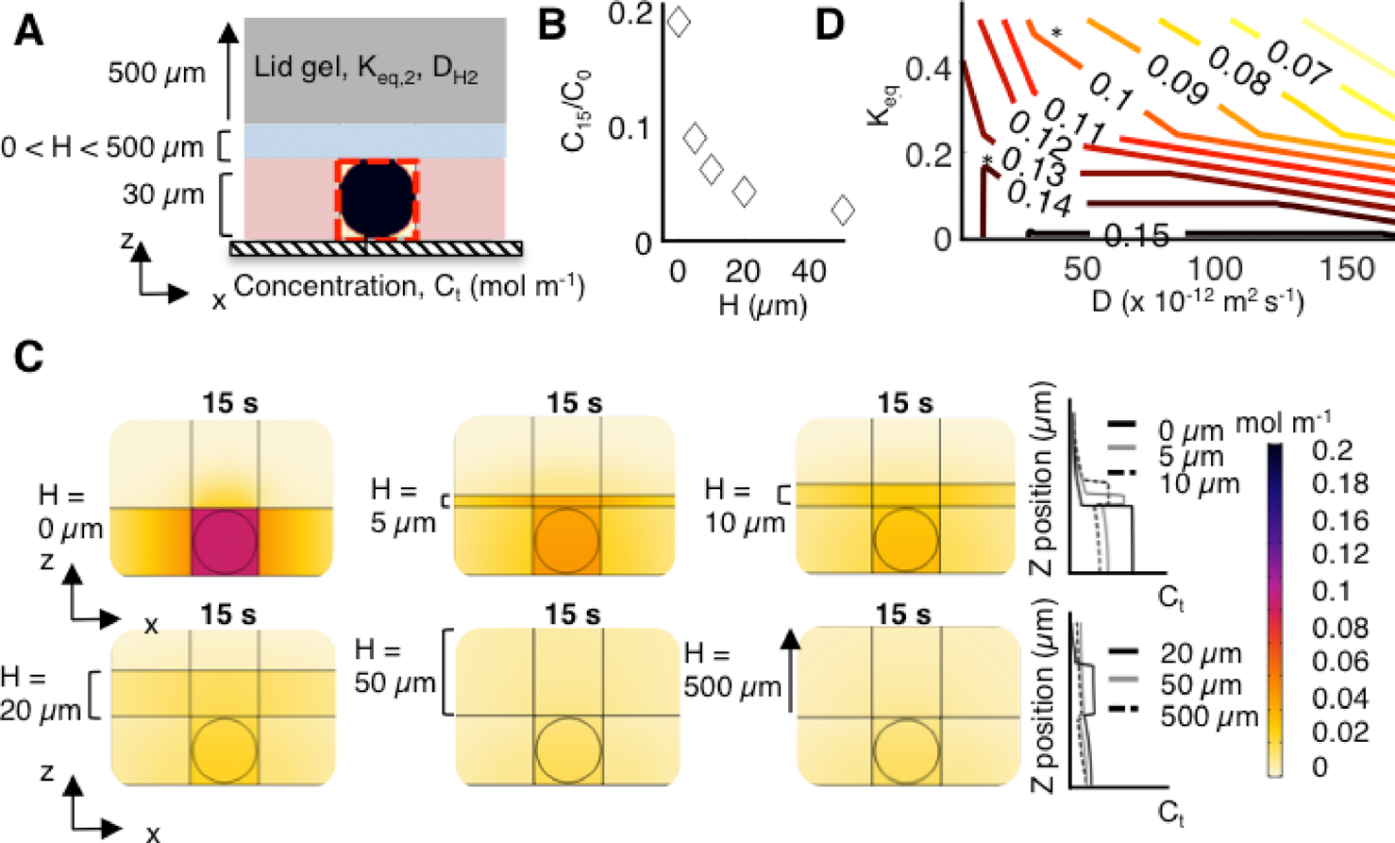
The protein solute concentration in the microwell depends on *H*, *K_eq_*, and *D* for GFP in various materials, as determined by numerical simulations. (a) Side-view schematic of the model for a range of *H* values with a lid gel. *H* was varied from 0 to 500 *μ*m. (b) Sensitivity analysis of the protein solute concentration in the microwell at time t = 15 s as a function of H, maintaining constant *D* and *K_eq_* values. As *H* increases, *C_t_*/*C*_0_ decreases from 0.20 to 0.09, 0.06, 0.04, and 0.03, for *H *=* *0, 5, 10, 20, and 50 *μ*m, respectively. At *H *=* *20 *μ*m, the change in *C_t_*/*C*_0_ as a function of *H* decreases to 0.03, indicating that the fluid film begins to act as a semi-infinite medium. (c) Simulation results show rapid dilution and diffusive losses of GFP with increasing *H*. (d) Contour plot of *C_t_*/*C*_0_ at time t = 15 s as a function of *K_eq_* and *D* for GFP given *H *=* *2 *μ*m. The asterisk indicates conditions where *D* and *K_eq_* for GFP are consistent with the material.

Given the simulation results indicating a high local concentration of proteins in the fluid film, we next sought to assess how *H*, *K_eq_*, and *D* affect the protein concentration in the microwell fluid volume [Fig. 4(a)]. We first varied the thickness of the fluid film (5, 10, 20, 50, and 500 *μ*m) and observed that, as the fluid film thickness increases, *C_t_*/*C*_0_ decreases from 0.20 to 0.09, 0.06, 0.04, and 0.03, respectively, at t = 15 s [Fig. 4(b)].

We noted a critical fluid film thickness, with regard to the protein concentration in the fluid film. At *H *=* *20 *μ*m, *C_t_*/*C*_0_ = 0.03 and increasing the *H* beyond 20 *μ*m has a muted effect on further reduction in *C_t_*/*C*_0._ In this geometry, we surmise that the fluid film begins to behave as a semi-infinite medium at *H *>* *20 *μ*m. This effect can be further seen when regarding the concentration profile from the XZ plane [Fig. 4(c)]. For *H *=* *20 *μ*m, the highest concentration of protein at t = 15 s is in the fluid film, whereas for *H *=* *0 *μ*m, the highest concentration of protein is in the microwell fluid volume. Within the microwell fluid volume, the maximum observed concentration of protein for *H *=* *20 *μ*m is 61% lower than that for *H *=* *0 *μ*m.

Comparatively, for *H *=* *500 *μ*m, the concentration of protein within the microwell fluid volume is 74% lower than that for H = 0 *μ*m. We conclude that the solute concentration in the microwell fluid volume and in the bulk of the bottom gel are sensitive to the fluid film thickness, especially when that thickness is smaller than 20 *μ*m for the configurations studied here [Fig. 1(b)]. Consequently, design strategies to minimize the fluid film thickness are critical in applications where maintaining a high concentration of solute in the microwell fluid volume is necessary.

Next, we sought to understand if *C_t_*/*C*_0_ is more sensitive to the partitioning effect of the lid or to the change in *D* of the GFP in the lid material. Previous studies point to the hydrogel composition (%T) having a greater influence on *K_eq_* than on *D* for proteins bovine serum albumin (BSA) and RNAse.^1^ We simulated *K_eq_* and *D* for GFP in different materials: liquid, the bottom gel, the lid gel, and glass. We assumed a 2-*μ*m fluid film thickness [Fig. 4(d)]. We calculated *C_t_*/*C*_0_ (t = 15 s) in the microwell fluid volume for each combination of *K_eq_ and D*. The simulation data reveal that *C_t_*/*C*_0_ is sensitive to both *K_eq_* and *D*, albeit with a different degree of sensitivity in different regimes. For low *K_eq_*, *C_t_*/*C*_0_ is relatively insensitive to *D* since the protein cannot partition into the lid gel. However, as *K_eq_* increases, *C_t_*/*C*_0_ (t = 15 s) drops rapidly. To maintain a constant value of *C_t_*/*C*_0_ with increasing *K_eq_*, *D* must correspondingly decrease. Similarly, as *D* in the lid material increases, *K_eq_* must decrease to maintain high *C_t_*/*C*_0._ Overall, the use of a lid composed of a dense gel is effective for high protein retention to the microwell fluid volume [asterisk on Fig. 4(d)]. Nonetheless, we can imagine further engineering material properties, such as decreasing both *D* and *K_eq_* via surface treatments^58^ and/or decreasing the effective PA pore size to decrease *D* of the protein in the lid material.^1^

## CONCLUSION

Molecular transport through hydrogels has implications in cell and tissue engineering, drug delivery, and single-cell assays. Here, we used numerical simulations to determine how the fluid film thickness, partition coefficient, and diffusion coefficient of GFP in multiple materials (liquid, dense hydrogel, and glass) dictate the GFP concentration in microwells. We first measured the fluid film thickness between two hydrogels of different densities (%T). We found that the application of a low-permeability or impermeable lid layer mitigates diffusive losses of proteins from microwells. Further, we find that the microwell protein concentration is dependent on the fluid film thickness, the partition coefficient, and the diffusion coefficient. Overall, we generated a model that provides a framework for how time-dependent protein diffusion depends on operational parameters.

From our simulations, we determined that the protein concentration in microwells is sensitive to the fluid film thickness; thus, design strategies to minimize or eliminate fluid films could result in higher retention of protein in microwells. To minimize the fluid film thickness, hydrogel properties such as surface roughness, permeability, or elastic modulus could be manipulated.^59^ Elastic modulus of gels can be tuned by changing cross-linking density;^60^ however, consideration of how modulating this parameter affects other properties of the gel, such as molecular sieving, is necessary.

In addition to minimizing the fluid film, material properties of the lid hydrogel may be modulated to reduce the diffusion coefficient and/or the partition coefficient of the species of interest in the lid. For example, modulating protein interactions with the lid layer by altering the charge^16^ or hydrophilicity^44^ presents strategies to tune the partition coefficient. In-gel diffusivity can be tuned by incorporating other polymeric materials such as Poly(ethylene glycol) diacrylate (PEGDA) to form interpenetrating networks that decrease solute diffusivity.^61^ Overall, tuning of the hydrogel free volume, obstructions (i.e., rigid or mobile polymer chains), and interactions with proteins could potentially provide solutions to maintaining high protein concentrations in microwells or make molecular transport more favorable for the desired application.

## METHODS

### Reagents

Acrylamide/bis-acrylamide 30% (w/w) solution, ammonium persulfate (APS), N,N,N′,N′-tetramethylethylenediamine (TEMED), imidazole, sodium phosphate, sodium chloride, sodium hydroxide, sodium deoxycholate, sodium dodecyl sulfate, and Triton X-100 were obtained from Sigma-Aldrich. 10× Tris-glycine was obtained from Bio-Rad. Tris-HCl, pH 6.8 buffer was obtained from Teknova. PureProteome nickel magnetic microparticles, 10 *μ*m, were obtained from Millipore-Sigma. TBST was obtained from Cell Signaling Technologies. Methacryloxyethyl thiocarbamoyl rhodamine B was obtained from Polysciences. VA-086 was obtained from Wako. N-[3-[(3-benzoylphenyl)formamido]propyl]methacrylamide (BPMA) was obtained from Pharm-Agra Laboratories. Silicon wafers were obtained from WaferNet. SU-8 developer and photoresist (SU-8 3050) were obtained from Microchem. Recombinant Protein G with His Tag was obtained from Abcam and labeled in-house with Alexa Fluor 647 NHS ester (Life Technologies). 0.5 *μ*m rhodamine-microbeads (FluoSpheres) were obtained from Life Technologies. Gel-Slick was obtained from Lonza.

### Fabrication of rhodamine-labeled PA gels

“Bottom” PA gels (6 %T, 3.3 %C) were synthesized using 5 mM BPMA and 0.005% (w/v) methacryloxyethyl thiocarbamoyl rhodamine B. The precursor solution was degassed and pipetted between an acrylate-silanized microscope slide or the No. 1 coverslip and a Gel-Slick treated silicon wafer patterned with 40 *μ*m SU-8 features, as previously described.^34^ PA gels were crosslinked using 0.08% (w/v) APS as the initiator and 0.08% (v/v) TEMED as the catalyst. After 20 min of polymerization, the gels were peeled off the wafer, rinsed with de-ionized water, and gently dried under a nitrogen stream or stored in 1× TBST solution. 15 %T, 3.3 %C lid gels containing 0.005% (w/v) methacryloxyethyl thiocarbamoyl rhodamine B were fabricated using photopolymerization, as previously described.^6^ The 500 *μ*m thickness of the high density 15 %T gels was obtained by patterning the gel between two glass plates separated by a 500 *μ*m thick spacer (CBS Scientific).

### Image acquisition

Confocal imaging experiments were conducted on an inverted Zeiss LSM 710 AxioObserver (Zeiss, Oberkochen, Germany). Images were acquired at room temperature using a 40× water objective (LD C-Apochromat 40×/1.1 NA W Corr M27, Zeiss) with the correction collar set for a No. 1 coverslip. Rhodamine-labeled PA gels were fabricated on No. 1 coverslips and imaged using a DPSS-561 laser at 0.25% power, using the MBS488/561/633 beam splitter and the Zen 2010 software (Zeiss). Z-stack images were acquired with a step size of 0.42 *μ*m with line scanning at x = y=z = 0.42 *μ*m pixel size.

Widefield epifluorescence images for microparticle imaging were obtained on an Olympus IX-71 inverted microscope with an Olympus LCPlanFI 40×/0.6 NA) objective and an EMCCD Camera iXon2 (Andor). For microparticle imaging, brightfield microscopy was first utilized to find the field of view including a microparticle in a microwell. Microparticles were then imaged with an exposure time of 50 ms using a Cy5 filter cube (Chroma, 49009) using a time series feature in MetaMorph (Molecular Devices). Images were collected every 1 s. For particle tracking, microparticles were imaged using an Olympus UPlanFi 10×/NA 0.3 objective at an exposure time of 500 ms and an EMCCD Camera iXon2 (Andor).

### Numerical simulations

Mass transport of proteins during cell lysis was simulated in COMSOL Multiphysics 5.3. Input parameters were obtained from the literature or were experimentally determined. Partition coefficients for GFP were 0.51, 0.24, and 0.10 for 6 %T PA gel to free solution, 15 %T PA gel to free solution, and 6 %T PA gel to 15 %T PA gel, respectively.^6^ The gel lid was 500 *μ*m in height, the bottom gel was 30 *μ*m in height, and the microwell was 30 *μ*m in height and width. To model an infinitely extending bottom gel, the widths of the bottom layer, fluid layer, and top layer were 10 000 *μ*m. These geometries were inputted into a 2D axisymmetric model. The maximum and minimum mesh element sizes were 30 and 0.3 *μ*m, respectively. Initial conditions were modeled in the Transport of Dilute Species module as a uniform starting concentration of GFP in a 28-*μ*m diameter spherical cell, comprising mostly liquid (liquid). The partition and diffusion coefficients of GFP in the cell were thus assumed to be those of liquid (*K_eq_*_,L_ = 1 and *D_L_* = 1.691 × 10^–10^ m^2^ s^−1^). The diffusivity of GFP was 4.2 × 10^–12^ m^2^ s^−1^, 3.13 × 10^–11^ m^2^ s^−1^, and 1.691 × 10^–10^ m^2^ s^−1^ in 15 %T gel, 6 %T gel, and liquid, respectively.^6^ The time step for lysis was 1 s. *C_t_*/*C*_0_ was estimated by taking a volume integral of the microwell at each 1 s interval and dividing by the volume integral of the microwell at t = 0 s.

For microparticle simulations, the same parameters as above were used. To simulate what would be measured via widefield microscopy, we included a 30-*μ*m wide rectangle directly above the microwell to include the fluid film and lid gel. For the microparticle, we first modeled the species of Protein G released from the microparticle and free to diffuse. We assumed that background-subtracted fluorescence intensity was equivalent to the number of fluorescent particles. The background-subtracted fluorescence intensity of the microparticle fits the function

$$y\left(t\right)=\;1135.8\;\exp\left(-0.037t\right).$$ (3)

This fluorescence as a function of time was assumed to be the amount of protein “bound” to the microparticle. For the free species, the fluorescence was calculated as the difference between *C*_0_ and *C_t_*. The flux, which represents the amount of free protein leaving the microparticle over the surface area per unit time, of the microparticle was calculated using the equation

$$N_{\mathrm{free}}\left(t\right)=\frac{d\left(y\left(0\right)-y\left(t\right)\right)}{\mathrm{dt}}\;\times \frac{1}{4\pi r^{2}}.$$ (4)

An exponential was fit to N(t), yielding

$$N\left(t\right)=\;44.425\;\exp\left(-0.037t\right).$$ (5)

The final concentration was determined by taking a volume integral of the free species over the surface of the microwell, including the column directly above the microwell encompassing the fluid layer and lid gel and summing to the measured fluorescence [y(t)] of the microparticle. The summed values were then normalized to the initial summed value.

Concentration profiles for the XZ and XY planes were obtained using the linear projection operator in COMSOL. To simulate the region surrounding a microwell, a rectangle of 100 *μ*m × 70 *μ*m was utilized. For the contour plot, simulations were run with a 500 *μ*m thickness lid layer using partition coefficients for GFP in glass, lid gel (15 %T PA gel), and the bottom gel (6 %T PA gel). For each partition coefficient, the simulation was run with three different diffusion coefficients for GFP (liquid, the bottom gel, and free solution), for a total of nine combinations. The protein solute concentration in the microwell was then obtained at t = 15 s and normalized to the initial concentration (t = 0 s). These values were inputted into a contour plot in MATLAB (R2017a) using the function *contour*.

### Bead tracking in convective flow

Bead tracking was performed as previously described.^5,62^ Briefly, 0.5 *μ*m fluorescent beads (FluoSpheres) were diluted 1:50 000 in phosphate buffered saline (PBS). Beads were then pipetted onto hydrated bottom gels (6 %T PA gels conjugated to glass slides). A lid gel (15 %T PA gel) was then interfaced to the bottom gel while imaging at an exposure time of 500 ms with an image acquisition rate of six frames per second. The velocity of the bead was quantified by measuring the length of the streak lines caused by the movement of the microspheres over the exposure period, as described previously.^5^ These values were compared with a negative control (no advection, no lid, and no pouring), which resulted in nondetectable velocities (no streaks). The Péclet number was calculated as

$$\mathrm{Pe}=L\frac{u}{D}$$ (6)

where L = 30 *μ*m, u is the average measured velocity of the bead, 285 *μ*m/s for lid gel or 13,000 *μ*m/s for pouring,^5^ and D = 1.691 × 10^–10^ m^2^ s^−1^, the diffusion coefficient of GFP in free solution.^6^

### Image processing and analysis

Fluid film thicknesses were determined from confocal images in a method described by Kuypers *et al.*^37^ Briefly, the fluorescence intensity of the XZ profiles was first background subtracted. The thickness of the fluid film was obtained by determining the “half shoulder” points, i.e., finding the local minima and maxima and determining the halfway point. The thickness was calculated as the difference in the Z-position of the half shoulder points.

### Microparticle fluorescence quantification

Microparticle fluorescence was quantified using an in-house MATLAB script for segmentation of the microparticle. We used a Canny edge detection approach for segmentation of the microparticle. After determining the microparticle boundaries and generating a binary mask, the mask was applied to all images in the time sequence that was first background-subtracted. The fluorescence was measured as the sum of all intensity values in the mask region. For microwell quantitation, a brightfield image was first taken to determine a region of interest (ROI) encompassing the microwell. The ROI was then used to measure background-subtracted fluorescence for each image in the time series.

### Statistical analysis

Statistical analysis for the two-sample Kolmogorov-Smirnov test was performed using the *kstest*2 function in MATLAB. The experimental group was a vector of the mean of four separate trials, and the simulation group was a vector containing the simulation data. The null hypothesis was that the data in each vector were from the same continuous distribution.

### Ethics approval

No ethics approval was required for this work.

## SUPPLEMENTARY MATERIAL

See supplementary material for microparticle release kinetics and advection from lid gel placement. Figures S1 demonstrates His-tagged protein release from Ni-surface functionalized magnetic microparticles. Figure S2 shows fluorescent bead tracking introduced by placement of a lid gel.

## References

[1] J. Tong and J. L. Anderson , Biophys. J. 70, 1505 (1996). 10.1016/S0006-3495(96)79712-6 8785307 PMC1225077

[2] J. C. Giddings , *Unified Separation Science* ( John Wiley & Sons, New York, 1991).

[3] S. H. Gehrke , J. P. Fisher , M. Palasis , and M. E. Lund , Ann. New York Acad. Sci. 831, 179 (1997). 10.1111/j.1749-6632.1997.tb52194.x 9616711

[4] A. G. Ogston , Trans. Faraday Soc. 54, 1754 (1958). 10.1039/tf9585401754

[5] A. J. Hughes , D. P. Spelke , Z. Xu , C.-C. Kang , D. V. Schaffer , and A. E. Herr , Nat. Methods 11, 749 (2014). 10.1038/nmeth.2992 24880876 PMC4077215

[6] A. M. Tentori , K. A. Yamauchi , and A. E. Herr , Angew. Chem. Int. Ed. 55, 12431 (2016). 10.1002/anie.201606039 PMC5201312 27595864

[7] A. Shekaran , J. R. García , A. Y. Clark , T. E. Kavanaugh , A. S. Lin , R. E. Guldberg , and A. J. García , Biomaterials 35, 5453 (2014). 10.1016/j.biomaterials.2014.03.055 24726536 PMC4033404

[8] J. D. Boerckel , Y. M. Kolambkar , K. M. Dupont , B. A. Uhrig , E. A. Phelps , H. Y. Stevens , A. J. García , and R. E. Guldberg , Biomaterials 32, 5241 (2011). 10.1016/j.biomaterials.2011.03.063 21507479 PMC3129848

[9] R. Dembczynski and T. Jankowski , Biochem. Eng. J. 6, 41 (2000). 10.1016/S1369-703X(00)00070-X 10908867

[10] B. Amsden , Macromolecules 31, 8382 (1998). 10.1021/ma980765f

[11] B. A. Westrin , A. Axelsson , and G. Zacchi , J. Controlled Release 30, 189 (1994). 10.1016/0168-3659(94)90025-6

[12] R. Schulz , K. Yamamoto , A. Klossek , R. Flesch , S. Hönzke , F. Rancan , A. Vogt , U. Blume-Peytavi , S. Hedtrich , M. Schäfer-Korting , E. Rühl , and R. R. Netz , Proc. Natl. Acad. Sci. 114, 3631 (2017). 10.1073/pnas.1620636114 28320932 PMC5389326

[13] R. Schwarzl , F. Du , R. Haag , and R. R. Netz , Eur. J. Pharm. Biopharm. 116, 131 (2017). 10.1016/j.ejpb.2016.12.015 28017797

[14] M. H. Hettiaratchi , A. Schudel , T. Rouse , A. J. García , S. N. Thomas , R. E. Guldberg , and T. C. McDevitt , APL Bioeng. 2, 026110 (2018). 10.1063/1.4999925 PMC6324205 31069307

[15] J. Siepmann , M. J. Rathbone , and R. A. Siegel , *Fundamentals and Applications of Controlled Release Drug Delivery* ( Springer US, New York, 2012).

[16] X. Zhang , J. Hansing , R. R. Netz , and J. E. DeRouchey , Biophys. J. 108, 530 (2015). 10.1016/j.bpj.2014.12.009 25650921 PMC4317548

[17] J. Hansing and R. R. Netz , Biophys. J. 114, 2653 (2018). 10.1016/j.bpj.2018.04.041 29874615 PMC6129561

[18] S. S. Jang , W. A. Goddard , and M. Y. S. Kalani , J. Phys. Chem. B 111, 1729 (2007). 10.1021/jp0656330 17249716

[19] J. D. Andrade , R. N. King , and D. E. Gregonis , *Hydrogels for Medical and Related Applications* ( American Chemical Society, 1976), pp. 206–224.

[20] X. Larrea and P. Büchler , Invest. Opthalmol. Visual Sci. 50, 1076 (2009). 10.1167/iovs.08-2479 18836169

[21] G. N. Orsborn and S. G. Zantos , CLAO J. 14, 81 (1988). 2456170

[22] J. J. Nichols and P. E. King-Smith , Invest. Opthalmol. Visual Sci. 44, 68 (2003). 10.1167/iovs.02-0377 12506057

[23] K. T. Sapra and H. Bayley , Sci. Rep. 2, 848 (2012). 10.1038/srep00848 23152939 PMC3497031

[24] J. J. Nichols and P. E. King-Smith , Invest. Opthalmol. Visual Sci. 45, 2549 (2004). 10.1167/iovs.04-0149 15277476

[25] A. M. Klein , L. Mazutis , I. Akartuna , N. Tallapragada , A. Veres , V. Li , L. Peshkin , D. A. Weitz , and M. W. Kirschner , Cell 161, 1187 (2015). 10.1016/j.cell.2015.04.044 26000487 PMC4441768

[26] A. Rotem , O. Ram , N. Shoresh , R. A. Sperling , A. Goren , D. A. Weitz , and B. E. Bernstein , Nat. Biotechnol. 33, 1165 (2015). 10.1038/nbt.3383 26458175 PMC4636926

[27] J. C. Love , J. L. Ronan , G. M. Grotenbreg , A. G. van der Veen , and H. L. Ploegh , Nat. Biotechnol. 24, 703 (2006). 10.1038/nbt1210 16699501

[28] T. M. Gierahn , M. H. Wadsworth , T. K. Hughes , B. D. Bryson , A. Butler , R. Satija , S. Fortune , J. C. Love , and A. K. Shalek , Nat. Methods 14, 395 (2017). 10.1038/nmeth.4179 28192419 PMC5376227

[29] Y. Lu , J. J. Chen , L. Mu , Q. Xue , Y. Wu , P.-H. Wu , J. Li , A. O. Vortmeyer , K. Miller-Jensen , D. Wirtz , and R. Fan , Anal. Chem. 85, 2548 (2013). 10.1021/ac400082e 23339603 PMC3589817

[30] A. J. Torres , A. S. Hill , and J. C. Love , Anal. Chem. 86, 11562 (2014). 10.1021/ac4030297 25347613 PMC4255675

[31] Q. Song , Q. Han , E. M. Bradshaw , S. C. Kent , K. Raddassi , B. Nilsson , G. T. Nepom , D. A. Hafler , and J. C. Love , Anal. Chem. 82, 473 (2010). 10.1021/ac9024363 20000848 PMC2828941

[32] H. Zhu , G. Stybayeva , M. Macal , E. Ramanculov , M. D. George , S. Dandekar , and A. Revzin , Lab Chip 8, 2197 (2008). 10.1039/b810244a 19023487

[33] Q. Shi , L. Qin , W. Wei , F. Geng , R. Fan , Y. S. Shin , D. Guo , L. Hood , P. S. Mischel , and J. R. Heath , Proc. Natl. Acad. Sci. U. S. A. 109, 419 (2012). 10.1073/pnas.1110865109 22203961 PMC3258586

[34] C.-C. Kang , K. A. Yamauchi , J. Vlassakis , E. Sinkala , T. A. Duncombe , and A. E. Herr , Nat. Protoc. 11, 1508 (2016). 10.1038/nprot.2016.089 27466711 PMC5511750

[35] C.-C. Kang , J.-M. G. Lin , Z. Xu , S. Kumar , and A. E. Herr , Anal. Chem. 86, 10429 (2014). 10.1021/ac502932t 25226230 PMC4204918

[36] J. Vlassakis and A. E. Herr , Anal. Chem. 89, 12787 (2017). 10.1021/acs.analchem.7b03096 29110464

[37] L. C. Kuypers , W. F. Decraemer , J. J. Dirckx , and J.-P. Timmermans , J. Microsc. 218, 68 (2005). 10.1111/j.1365-2818.2005.01457.x 15817065

[38] G. McClure , Z. M. Jin , J. Fisher , and B. J. Tighe , Proc. Inst. Mech. Eng., Part H 210, 89 (1996). 10.1243/PIME_PROC_1996_210_397_02 8688121

[39] A. J. Engler , S. Sen , H. L. Sweeney , and D. E. Discher , Cell 126, 677 (2006). 10.1016/j.cell.2006.06.044 16923388

[40] L. L. Ting , Wear 34, 159 (1975). 10.1016/0043-1648(75)90062-9

[41] N. M. Bujurke and H. P. Patil , Int. J. Mech. Sci. 34, 355 (1992). 10.1016/0020-7403(92)90023-A

[42] K. A. Yamauchi and A. E. Herr , Microsyst. Nanoeng. 3, 16079 (2017). 10.1038/micronano.2016.79 29333327 PMC5764185

[43] E. J. Su and A. E. Herr , Lab Chip 17, 4312 (2017). 10.1039/C7LC01012E 29120467 PMC6275091

[44] G. Cevc , Eur. J. Pharm. Biopharm. 92, 204 (2015). 10.1016/j.ejpb.2015.03.008 25794476

[45] J. J. Kim , P. P. Y. Chan , J. Vlassakis , A. Geldert , and A. E. Herr , Small 14 1802865 (2018). 10.1002/smll.201802865 PMC6272123 30334351

[46] B. Voit , F. Braun , M. Gernert , B. Sieczkowska , M. Millaruelo , M. Messerschmidt , M. Mertig , and J. Opitz , Polym. Adv. Technol. 17, 691 (2006). 10.1002/pat.793

[47] F. Braun , L. Eng , S. Trogisch , and B. Voit , Macromol. Chem. Phys. 204, 1486 (2003). 10.1002/macp.200350015

[48] F. Braun , L. Eng , C. Loppacher , S. Trogisch , and B. Voit , Macromol. Chem. Phys. 203, 1781 (2002).

[49] Y. Wu , L. Zhang , M. Zhang , Z. Liu , W. Zhu , and K. Zhang , Polym. Chem. 9, 1799 (2018). 10.1039/C8PY00182K

[50] N. De Alwis Watuthanthrige , P. N. Kurek , and D. Konkolewicz , Polym. Chem. 9, 1557 (2018). 10.1039/C7PY01398A

[51] D. Shi , M. Matsusaki , and M. Akashi , J. Controlled Release 149, 182 (2011). 10.1016/j.jconrel.2010.08.009 20727923

[52] Q. Jin , F. Mitschang , and S. Agarwal , Biomacromolecules 12, 3684 (2011). 10.1021/bm2009125 21863834

[53] B. S. Schuster , D. B. Allan , J. C. Kays , J. Hanes , and R. L. Leheny , J. Controlled Release 260, 124 (2017). 10.1016/j.jconrel.2017.05.035 PMC6037165 28578189

[54] A. V. Kabanov and S. V. Vinogradov , Angew. Chem. Int. Ed. 48, 5418 (2009). 10.1002/anie.200900441 PMC2872506 19562807

[55] X. Yuan , K. Fischer , and W. Schärtl , Langmuir 21, 9374 (2005). 10.1021/la051491+ 16171376

[56] J. Jiang , X. Tong , D. Morris , and Y. Zhao , Macromolecules 39, 4633 (2006). 10.1021/ma060142z

[57] S. R. Mujumdar , R. B. Mujumdar , C. M. Grant , and A. S. Waggoner , Bioconjugate Chem. 7, 356 (1996). 10.1021/bc960021b 8816960

[58] K. J. Seu , A. P. Pandey , F. Haque , E. A. Proctor , A. E. Ribbe , and J. S. Hovis , Biophys. J. 92, 2445 (2007). 10.1529/biophysj.106.099721 17218468 PMC1864818

[59] A. C. Dunn , W. G. Sawyer , and T. E. Angelini , Tribol. Lett. 54, 59 (2014). 10.1007/s11249-014-0308-1

[60] J. R. Tse and A. J. Engler , Curr. Protoc. Cell Biol. 47, 10.16.1 (2010). 10.1002/0471143030.cb1016s47 20521229

[61] Y. Fu and W. J. Kao , Pharm. Res. 26, 2115 (2009). 10.1007/s11095-009-9923-1 19554430 PMC3809113

[62] T. A. Duncombe and A. E. Herr , Lab Chip 13, 2115 (2013). 10.1039/c3lc50269d 23609800

